# Reforming China’s Secondary Vocational Medical Education: Adapting to the Challenges and Opportunities of the AI Era

**DOI:** 10.2196/48594

**Published:** 2024-08-15

**Authors:** Wenting Tong, Xiaowen Zhang, Haiping Zeng, Jianping Pan, Chao Gong, Hui Zhang

**Affiliations:** 1 Department of Pharmacy, Gannan Healthcare Vocational College, Ganzhou, China; 2 Department of Rehabilitation and Elderly Care, Gannan Healthcare Vocational College, Ganzhou, China; 3 Department of Gastrointestinal Surgery, Guangdong Provincial Hospital of Chinese Medicine, Guangzhou, China; 4 Department of Gastrointestinal Surgery, First Affiliated Hospital of Guangzhou University of Chinese Medicine, Guangzhou, China; 5 Scientific Research Division, Gannan Healthcare Vocational College, Ganzhou, China; 6 Student Work Division, Gannan Healthcare Vocational College, Ganzhou, China; 7 Department of Infertility and Sexual Medicine, Third Affiliated Hospital of Sun Yat-sen University, Guangzhou, China; 8 Department of Urology, Dongguan Hospital Affiliated to Guangzhou University of Chinese Medicine, 22 Songshanhu Avenue, Guangdong Province, Dongguan, 523080, China, 86 0769 2638 5365

**Keywords:** secondary vocational medical education, artificial intelligence, practical skills, critical thinking, AI

## Abstract

China’s secondary vocational medical education is essential for training primary health care personnel and enhancing public health responses. This education system currently faces challenges, primarily due to its emphasis on knowledge acquisition that overshadows the development and application of skills, especially in the context of emerging artificial intelligence (AI) technologies. This article delves into the impact of AI on medical practices and uses this analysis to suggest reforms for the vocational medical education system in China. AI is found to significantly enhance diagnostic capabilities, therapeutic decision-making, and patient management. However, it also brings about concerns such as potential job losses and necessitates the adaptation of medical professionals to new technologies. Proposed reforms include a greater focus on critical thinking, hands-on experiences, skill development, medical ethics, and integrating humanities and AI into the curriculum. These reforms require ongoing evaluation and sustained research to effectively prepare medical students for future challenges in the field.

## Introduction

A well-established medical education system is pivotal in training a sufficient number of high-quality professionals to meet societal health needs and tackle challenges. China’s medical education structure encompasses secondary vocational medical education, undergraduate, master’s, and doctoral degrees [1]. Specifically, secondary vocational medical education is a 3-year program for junior high school graduates [2]. Its origin traces back to the “barefoot doctors” of the 1960s. While not all were formal doctors, they underwent fundamental medical and health training, primarily serving rural areas. To address the medical service shortage in rural areas, the Chinese government trained a group of farmers with basic medical skills in the 1960s [3,4]. These barefoot doctors played a pivotal role in China’s health care system, significantly alleviating rural medical service shortages and improving overall health standards. However, as the medical system and the economy evolved in the late 1970s and early 1980s, the barefoot doctor model gradually phased out [3]. Despite its development, China still exhibits a dual nature due to uneven progress. On one side, in economically developed coastal and major urban areas, medical resources are comparable with those in economically developed regions such as Europe and America. On the other side, similar to some regions in Asia or Africa, areas such as Qinghai, Tibet in the west of China and many rural locales experience a severe lack of medical resources. In some of these areas, the standards for practicing qualifications have even been lowered to meet basic health care needs. This stark contrast underscores the challenge of achieving equitable health care access across diverse geographic and economic landscapes [5-8].

Secondary vocational medical education can be seen as an evolved version of the barefoot doctor model, aiming to address medical resource shortages and service imbalances due to regional disparities [1]. The core objectives of this educational system are to enhance grassroots medical levels, nurture qualified medical personnel, and reinforce grassroots medical institution infrastructure, thus bolstering public health response capabilities [9]. Nevertheless, this system heavily relies on traditional teaching methods, leading to a significant disconnect between theory and practice for students [10].

Artificial intelligence (AI) is profoundly reshaping the medical sector, but the current secondary vocational medical education system has not fully integrated AI technology into its teaching. To ensure that students can fully harness and address these technological revolutions, educators need to reconsider curriculum design, integrating these cutting-edge technologies and preparing students for future medical innovations [11-13]. Based on the long-term experience accumulated by the team in secondary vocational medical education and existing research, this study will delve into the challenges faced by secondary vocational medical education in the era of AI and potential strategies to address them, and this analysis will provide valuable insights and lessons that can be applied to similar countries and regions at various levels of economic development.

## Opportunities and Challenges Faced by Secondary Vocational Education in the Era of AI

**Figure 1.**
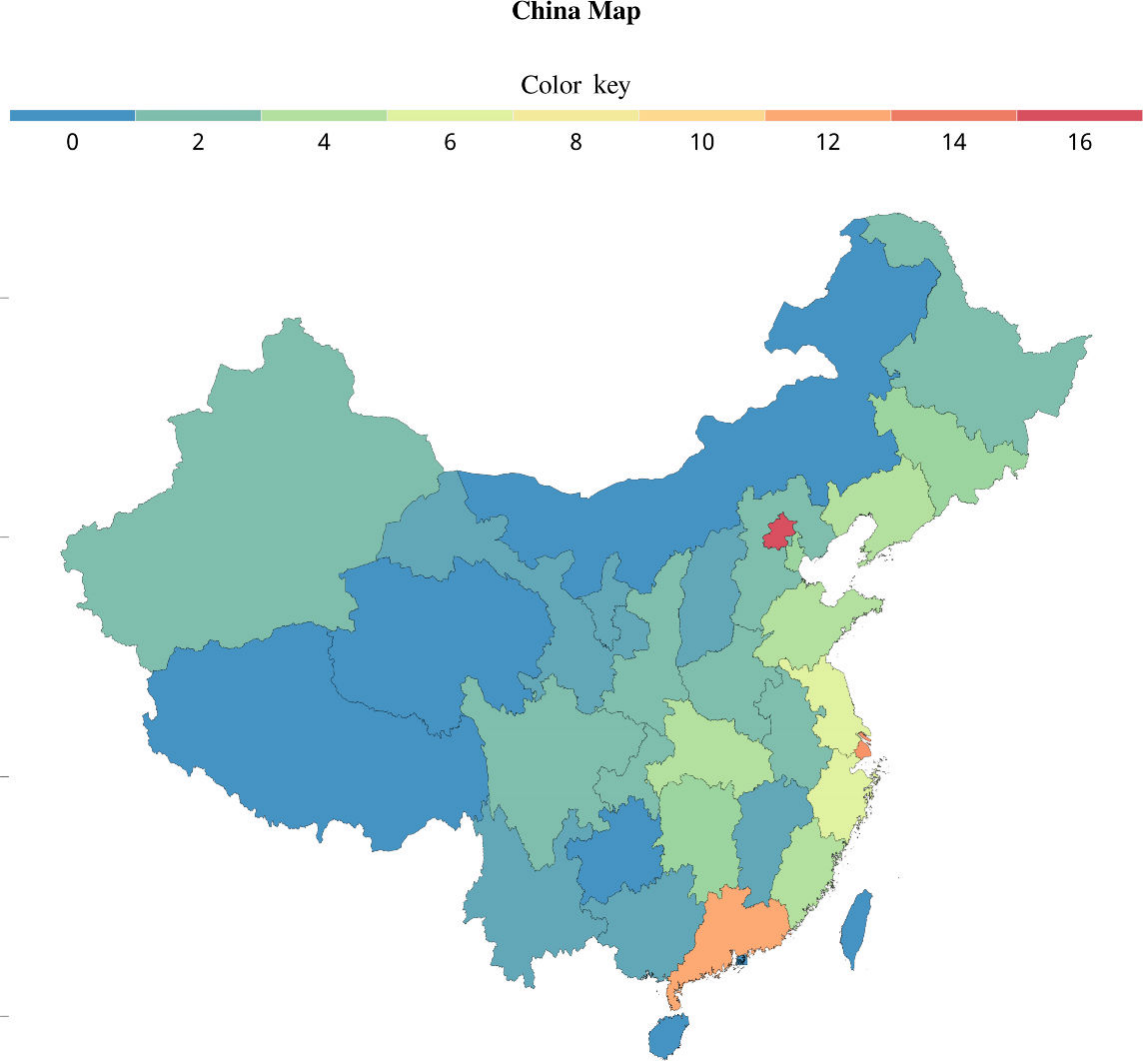
Distribution of the top 100 hospitals in China. Data were sourced from Fudan University [16]. Data from Taiwan, Hong Kong, and Macau are excluded from this analysis.

Over the past 70 years, the grassroots medical standard in China has been improving. However, constrained by economic development and population growth, the distribution of medical resources across the country remains limited and severely imbalanced. A small amount of high-quality medical resources is concentrated in economically developed areas (Figure 1), and medical staff generally bear a high workload. Research indicates that in rural grassroots areas, medical personnel work an average of about 8.9 hours daily, working at least 6 days a week [14]. Another study shows that in 2010, 2015, and 2016, health care workers had monthly workloads exceeding 30 days for 6, 5, and 9 months, respectively [15]. The massive patient flow due to the large number of patients places a heavy burden on doctors.

Concurrently, the 3-year fast-track training provided by secondary vocational medical education does not endow medical students with the plethora of skills they should ideally possess. Many doctors who enter grassroots work lack systematic and in-depth professional training. They might rely merely on basic medical knowledge and experience to diagnose and treat patients. While this may suffice for the majority of primary diagnoses and treatments, it still falls short when dealing with complex cases [17,18]. Combined with the heavy workload, this could potentially lead to misdiagnoses or overtreatment.

It is noteworthy that the overwhelming workload also results in doctors having little desire to communicate adequately with patients [19]. In many instances, the root cause of doctor-patient disputes is not merely the misdiagnosis itself but rather the lack of effective communication between the doctor and the patient.

In recent years, the rapid development of AI has demonstrated its potential impact on China’s health care landscape. In the realm of direct patient care, the application of AI not only has the potential to enhance the efficiency of medical services [20-26] but also opens new opportunities for medical equity across different regions [27].

Before the widespread application of generative AI, scientists had already been using AI imaging systems and other technologies to address the disparities in medical standards between regions. In primary health care settings, AI is progressively gaining prominence and is perceived as an auxiliary tool with immense potential [28-30]. Numerous studies have highlighted that particularly in areas lacking experienced radiologists, AI plays a pivotal role in medical imaging analysis, such as in x-rays or basic scans [31,32]. Furthermore, AI offers data-driven therapeutic suggestions not only to physicians, enhancing the accuracy and efficiency of treatments, but also in health care resource management, such as bed allocation. Its predictive models proficiently optimize resource allocation, ensuring that patients receive timely and appropriate care [12]. In the context of telemedicine, the integration of AI with wearable devices undoubtedly delivers more precise health information to medical practitioners, leading to more efficient health management. This would substantially alleviate the workload of health care providers, laying the groundwork for a more harmonious doctor-patient relationship [33].

Building on this foundation, generative AI technologies such as ChatGPT have opened up new possibilities for enhancing primary health care. Multiple studies have confirmed the significant potential and reliability of generative AI in the medical field, enhancing the decision-making capabilities of primary care physicians [20-24]. Notably, these technologies have also shown significant potential in improving doctors’ communication skills.

Communication is one of the core skills for physicians, especially when dealing with a large number of patients. Effective communication skills can aid in better patient recovery and provide a harmonious practice environment [34,35]. Studies have demonstrated that ChatGPT exhibits a high level of empathy when addressing common queries [22,36]. In addition, research has explored the potential of generative AI to enhance the communication skills of emergency medical doctors, particularly in delivering bad news, by simulating patient reactions and dialogues during the disclosure of a cancer diagnosis [37]. With the aid of AI, we can better simulate clinical environments, thus improving the training of medical students in patient communication [38].

Furthermore, the future of medical practice will become more complex, requiring doctors to not only possess professional knowledge but also have a basic understanding of technologies related to health care, such as blockchain, cloud services, data quality, machine learning, electronic health records, and mobile health. Some of these technologies are straightforward, while others are complex; AI can help beginners simplify concepts and accelerate their learning [39].

However, despite the conveniences and advantages that AI brings to the training of medical students in primary care, we must still face several inherent challenges: the threat to employment, the necessity of skill updates, and the ongoing need for training. The automation capabilities of AI may gradually replace some basic and repetitive tasks, such as preliminary diagnosis and data entry, which could impact the job stability of medical personnel [12,13].

Moreover, the rapid development of AI technology may increase the obsolescence of certain traditional medical practices and skills. To keep pace with technological progress, health care workers may face more frequent training demands. Although the concept of lifelong learning is inherently positive, it could impose additional psychological stress on doctors [40,41].

Advancements in technology, while opening new treatment possibilities, also raise new ethical issues, such as data privacy and machine bias, which need to be addressed and resolved in medical education [42]. In addition, while problem-based learning (PBL) approaches attempt to bridge the gap between theory and practice, a lack of practical opportunities and the disconnection between theory and practice might leave students feeling unprepared when facing real medical challenges. These challenges need to be carefully considered and overcome in the AI-integrated educational process to ensure the quality of education and the professional development of students.

At this stage, AI primarily acts as an auxiliary tool [43], helping medical personnel solve problems more effectively and optimize services to patients. As educators, we have a responsibility to directly address any fears students may have [44] and to start popularizing and applying AI knowledge from the educational phase. This will help them more effectively use these technologies in their future practice and reduce resistance to new technologies.

The reform of AI in education will not happen overnight, as school reforms often depend on policy support and tend to lag [45]. Furthermore, the development of AI requires the integration of technology, which in turn necessitates significant resource investment, such as in hardware, software, and professional talent. Unequal resource distribution could make achieving this goal difficult. The rapidly changing technological environment also demands frequent updates to educational content, presenting ongoing challenges for educational institutions.

## Recommendations for the Reform of Medical Education in Chinese Secondary Vocational Schools

### Integration With Technology

In recent years, AI has taken a central role amidst the technological revolution in the medical field, particularly given its significant impact on enhancing diagnosis and treatment efficiencies. To adapt to this trend, secondary vocational medical education must adjust to ensure that students not just grasp traditional medical techniques but also intertwine with AI technology and applications. This encompasses understanding the significance of machine learning algorithms and data analysis techniques, as well as how to effectively use AI in real-world medical settings [46].

The preclinical teaching phase serves as an ideal starting point. Strengthening courses on health data management, integration, and governance and emphasizing foundational AI, ethics, and legal issues are paramount [46,47]. These courses can be offered independently, ensuring that students maintain foundational knowledge even if certain technologies or applications become obsolete [48].

In addition, students should comprehend the computer and software engineering principles behind AI applications. Insights into hardware and software development and foundational knowledge of user experience design could be invaluable for their future medical careers [49-51]. Furthermore, during clinical rotations and residencies, students should focus on the practical application of AI, such as the widespread use of AI-based technologies for digital biomarkers and therapies in home settings, which offer large-scale diagnostic and therapeutic solutions [52,53]. In essence, tightly weaving AI into secondary vocational medical education will equip students to serve patients better, ensuring efficiency and accuracy in medical services.

### Lifelong Learning

Given the constantly evolving nature of medicine, it is imperative for practitioners to adapt continually to its changing landscape. To ensure that medical students thrive in this dynamic environment, there should be a heightened emphasis on cultivating a growth mindset and fostering lifelong learning capabilities. This mindset encourages viewing challenges and failures as opportunities for learning and growth rather than end points [54,55]. With rapid advancements in medical technology and treatments, students need the awareness and ability to continually refresh their knowledge and skills, keeping them current [56,57]. Offering students exposure and hands-on experience with AI tools not only aligns them with current medical technology trends but also instills a strong adaptive and continuous learning culture—a key to success in a fast-evolving medical field [58,59].

### Nurturing Ethical and Critical Thinking

There is a growing global focus on how medical curriculum design balances traditional medical education with the integration of emerging technologies [60-62]. Enhancing the medical curriculum should include not just traditional medical knowledge but also medical ethics and humanities [63,64]. Such a structure not just cultivates students’ grasp of medical concepts but also strengthens their ethical foundations, critical thinking, and decision-making abilities [65]. With advancements in medical technology, especially the widespread adoption of AI, students must learn to balance technology use and ethics. Despite the unparalleled conveniences AI offers in health care, it has evident limitations. Students need sound judgment to ensure optimal treatment choices for patients [66-68]. Furthermore, as AI’s role in medicine expands, solidifying foundational medical knowledge becomes even more crucial. Students require a robust medical foundation, providing them with a framework to make accurate judgments about AI technologies and ensuring their correct clinical application [69].

### Emphasizing Practical Experience

Modern medical education is at a pivotal juncture, necessitating a closer alignment of profound theoretical knowledge with actual medical practice. To achieve this, there is a need to revisit and optimize the curriculum, placing hands-on experience and skill cultivation at its core. PBL offers a direction for this educational transformation. PBL not only stimulates students’ proactivity, enabling them to devise solutions for real medical scenarios, but also nurtures their critical thinking abilities [70,71]. At the same time, the advent of AI will compel educators to abandon traditional teacher-centered instructional methods. With the assistance of AI, educators can facilitate active participation and personalized education for students. They can generate learning materials tailored to each student’s learning status and needs, such as by simulating standard patients, providing diverse case studies, and offering brainstorming activities and practice exercises [72,73], thereby increasing clinical internship opportunities that allow students to delve deeper into and comprehend medical practice. Specific practical activities, such as internships and simulated diagnostics, not only deepen students’ understanding of medical environments but also aid them in making wiser decisions when faced with intricate medical issues [74]. More crucially, such authentic clinical experiences bridge the gap between theoretical knowledge and practical operation, helping students foster a more professional demeanor, boosting their confidence, and ensuring superior performance in real medical settings [75].

### Resource Allocation

To ensure the successful implementation of medical education reform, it is essential to focus on optimizing resource distribution [76]. Specifically, financial investments should be concentrated on upgrading educational infrastructure, acquiring and maintaining new technologies, and establishing a dedicated fund to support the technologization of medical education. In addition, the professional development of teachers is crucial. Systematic training must be provided on AI and related technologies to ensure that teachers possess the most advanced knowledge and skills. Schools should also be equipped with the necessary technical resources to access the latest medical databases and AI tools, such as high-speed internet, updated computer hardware, and software.

### Policy Support

In terms of policy support, reforms in medical education should be facilitated through the development of policies that specifically support technology integration and lifelong learning. This includes establishing standards for educational quality and teacher evaluations while encouraging cross-sector collaboration between education departments and health, technology, and private sectors. Importantly, a comprehensive regulatory framework needs to be established to monitor the application of AI technology in medical education, ensuring that all activities comply with ethical and legal standards to protect the rights of students and patients.

### Infrastructure Development

Modernizing educational infrastructure is key to enhancing teaching quality [77,78]. Relevant authorities should invest in upgrading traditional classrooms and laboratories to support applications such as virtual reality and augmented reality. Combined with AI, these technologies can be used to simulate complex medical scenarios and surgical procedures [79]. Developing or adopting advanced learning management systems to support web-based teaching and resource sharing, as well as constructing more modern clinical training facilities, can significantly enhance students’ practical skills and lay a solid foundation for their future careers.

## Discussion

As noted in earlier sections of this paper, China’s secondary vocational medical education system, while comprehensive, still relies heavily on traditional teaching methods that emphasize rote memorization over practical application and critical thinking skills [80]. As we move further into the era of AI, these educational frameworks are becoming increasingly outdated. Technologies such as ChatGPT offer the potential to radically reform these traditional systems. By using AI tools and methods, we can address many of the current issues in our educational system, such as the disconnect between theoretical knowledge and practical application.

In the AI era, medical students are presented with unique opportunities to access a wealth of medical curricula previously unimaginable. For instance, AI integrated with augmented reality and virtual reality can greatly enhance interactivity, creating more engaging learning environments that allow students to practice skills in a risk-free setting [79]. Moreover, AI can facilitate personalized education, adjusting learning materials and pacing to meet individual student needs [72,73]. By leveraging these technologies, educational institutions can cultivate more skilled and versatile medical professionals who are well prepared to tackle the challenges of the modern medical environment.

Within the context of China’s secondary vocational medical education, practical applications of AI should include the introduction of AI-driven diagnostic tools during clinical rotations, allowing students firsthand experience with their use. This exposure not only enhances their diagnostic capabilities but also enables them to critically understand the advantages and limitations of AI-assisted decision-making [81,82].

To effectively integrate AI into medical education, educational departments must revise curricula to include specialized courses in data science, machine learning, and the ethical considerations of using AI [63,64]. These courses should be designed to ensure students comprehend both the capabilities and limitations of AI technology. In addition, training for educators must also be undertaken to ensure that they possess the requisite up-to-date knowledge and concepts to teach these new modules.

While the benefits of integrating AI into medical education are clear, significant challenges and potential resistance exist. These challenges include transforming traditional educational paradigms, the high costs of technological integration, and the need for continual curriculum updates to keep pace with technological advancements. A crucial step in addressing these challenges involves engaging all stakeholders—including educators, students, and policy makers—in the educational reform process. Demonstrating the specific benefits of AI in enhancing student learning outcomes and patient care can help garner broader support to realize these changes.

## Conclusions

The future of medical education in China, particularly at secondary vocational schools, will largely depend on the ability of educators, policy makers, and society to adapt and respond to technological advances. By embracing AI and incorporating it into curriculum design, we can train the next generation of health care professionals, equipping them not only with traditional medical knowledge but also with the skills to use technology to improve patient outcomes. Although challenges exist in the reform process, it is vital to ensure that medical students are well prepared for future medical practices.

## Abbreviations

AI: artificial intelligence
PBL: problem-based learning
